# Insect functional traits reveal processes that shape niche differentiation patterns

**DOI:** 10.1007/s00442-025-05783-4

**Published:** 2025-08-21

**Authors:** Robert Grosdidier, Raelene M. Crandall, Emma Silverman, Philip G. Hahn

**Affiliations:** 1 Entomology and Nematology Department, University of Florida, Gainesville, FL USA; 2 School of Forestry, Fisheries, and Geomatics Sciences, University of Florida, Gainesville, FL USA; 3 School of Natural Resources and Environment, University of Florida, Gainesville, FL USA; 4 Present Address: Department of Entomology, Purdue University, West Lafayette, IN USA

**Keywords:** Community assembly, Environmental filtering, Feeding niche, Grasshopper, Insect traits

## Abstract

Functional traits allow ecologists to infer processes that structure ecological communities and quantify interactions between trophic levels. Insects are a ubiquitous group that exhibits a variety of functional traits, and studying these traits could prove useful for understanding insect community assembly. We use insect trait measurements, community sampling along a disturbance gradient, and a cafeteria-style feeding assay to elucidate the mechanisms that govern insect herbivore community assembly processes. Using a trait principal component analysis, we first identified life history trade-offs between herbivore feeding and nutrition based on 3 traits measured on 14 grasshopper species collected across 30 pine savanna field sites. In the field, we tested hypotheses regarding how competition or environmental filtering might structure communities by examining dispersion patterns of functional traits. We found that grasshopper incisor strength and C:N ratio were consistently under-dispersed, patterns that indicate trait-clustering resulting from environmental filtering. Using mesocosms, we tested the hypothesis that grasshopper species should exhibit differentiation in their feeding niches based on the traits of the plants they consume and that feeding traits should correlate with traits of consumed plants. We demonstrated that grasshopper species strongly differentiated their feeding niche based on the traits of plants incorporated into their diets. We also found linkages between herbivore incisor strength and plant leaf dry matter content, and between grasshopper C:N ratio or body size and plant C:N ratio. This study revealed likely mechanisms that govern the interactions between insect herbivores, the plants they feed on, and how they partition resources to coexist.

**Supplementary Information:**

The online version contains supplementary material available at 10.1007/s00442-025-05783-4.

## Introduction

For over a century, ecologists have explained the assembly of diverse communities through niche-based processes, where species evolve different strategies to partition resources and minimize competition (Gause 1934; Hutchinson 1957; James 1971). More recently, ecologists have begun examining functional traits to elucidate niche-based mechanisms structuring communities (Chase and Leibold 2003; McGill et al. 2006; Violle et al. 2007). Functional traits are any morpho-, physio-, or phenological traits that impact an organism’s fitness (Violle et al. 2007). Functional traits gained attention from community ecologists around the turn of the century when researchers highlighted the predictive power of plant traits for explaining community processes (Weiher and Keddy 1995; Lavorel and Garnier 2002). For example, functional traits have shown that plant communities shift from slower-growing, more stress-tolerant species to faster-growing, more acquisitive species along gradients of increasing resource availability (Wright et al. 2004; Cornwell and Ackerly 2009; Reich 2014). Plant ecologists have widely used traits to understand these processes (Funk et al. 2017), but less attention has been paid to other taxa, such as insects, despite their ubiquity and the fact that they exhibit a wide range of functional trait values linked to important ecological processes (Wong et al. 2019).

Insect functional traits could shed light on how generalist herbivores can coexist within a community despite utilizing similar food resources. For example, grasshoppers, which are diverse and dominant generalist herbivores in grassland ecosystems (Branson et al. 2006; Cease et al. 2012), exhibit overlapping diets within communities (Joern 1979; Chase 1996). Previous laboratory feeding experiments have shown that grasshoppers differentiate niches based on the ratios of proteins and carbohydrates incorporated into their diet (Behmer and Joern 2008). These patterns of diet specialization may promote niche differentiation and coexistence under field conditions, but this has not yet been tested. Differentiated functional trait values likely mediate this diet specialization between grasshopper species (Ibanez et al. 2013). For example, if an insect is to feed on some plant species, it must possess the necessary mandible strength and metabolic capacity to break down the plant material. In turn, the plant’s toughness and the carbon-to-nitrogen ratio must meet the insect’s physiological requirements. Body size is a common insect functional trait that has been shown to be related to metabolic rates that can impact ecosystem functioning (Ibanez 2012; Garibaldi et al. 2015; Le Provost et al. 2017; Brousseau et al. 2018). For example, a large body size in insects has been correlated with increased foraging behavior which subsequently increases the abundance and distribution of the species (Friess et al. 2017; Pinkert et al. 2020). Further, traits are often highly correlated due to physiological constraints dictated by life history trade-offs (Flatt and Heyland 2011). Clearly, insect functional traits have the potential to impact community processes, and exploring the role of individual traits and their interaction with other traits could provide a more mechanistic understanding of insect community assembly.

Examining co-occurrence patterns of functional traits in the field can shed light on how competition or abiotic filtering structures communities. Community assembly theory posits that four main forces shape community structure: ecological selection (i.e., environmental filtering or competition), dispersal, drift, and speciation (Vellend 2010). Functional traits can provide inference into drivers of ecological selection, specifically differentiating between environmental filtering and competition (Weiher and Keddy 1995). Ecological selection driven by strong competition among species would exhibit a pattern of trait over-dispersion, where similar species would outcompete each other and species with dissimilar traits and niches would coexist (Cavender‐Bares et al. 2009). In contrast, ecological selection driven by environmental filters would exhibit clustering of functional traits within a community and lead to trait under-dispersion. Trait clustering occurs because specific environmental conditions select species with similar traits and niches (Cavender‐Bares et al. 2009). Interpreting trait dispersion patterns from observational data is notoriously difficult (Kraft et al. 2015) and is most informative when combined with additional data or experiments (Cadotte and Tucker 2017; Wong et al. 2019).

In grasslands and savannas, such as the pine savannahs in the southeastern USA, fire is a common disturbance that strongly shapes the taxonomic and functional structure of plant communities (Mitchell et al. 2021). Prior to European colonization, pine savannas were a dominant ecosystem throughout much of the southern Atlantic Coast and eastern Gulf Coast, ranging from Virginia to northern Florida and as far west as eastern Texas (Glitzenstein et al. 1995; Jose et al. 2006). The ecosystem ranges from xeric sandhills to poorly drained, mesic flatwoods (Glitzenstein et al. 1995; Jose et al. 2006). Pine savannas are fire-dependent ecosystems, with many species having adapted to the frequent occurrence of wildland fires (Glitzenstein et al. 1995; Jose et al. 2006). The range of habitat characteristics and human-mediated prescribed fire regiments in pine savannas allows for a diverse community of flora and insect fauna, making pine savannas a suitable system for studying the dynamics of plant and insect communities.

Grasshoppers (Orthoptera: Acrididae) represent a diverse taxa of insect herbivores in pine savanna ecosystems (Hahn and Orrock 2015a) and exhibit a variety of functional traits related to feeding niche, dispersal, and behavior (Deraison et al. 2015a; Bakewell et al. 2020). Considerable research has established that grasshoppers have important impacts on the functioning of grassland ecosystems through herbivory (Rodell 1977; Belovsky and Slade 2018), nutrient cycling by converting plant material to frass (Belovsky and Slade 2000), and as prey for higher trophic levels (Belovsky and Slade 1993). A primary goal of this study was to determine if grasshopper traits are linked to the traits of plants they consume and whether grasshopper species differentiate their feeding niches based on plant functional traits. Establishing these trait correlations is known as ‘trait-matching,’ a tool used to identify functional linkages between trophic levels (Garibaldi et al. 2015; Wong et al. 2019). If functional traits mediate linkages between trophic levels, then correlations should exist between the trait values of insect herbivores and those of plants on which they preferentially feed.

**Fig. 1:**
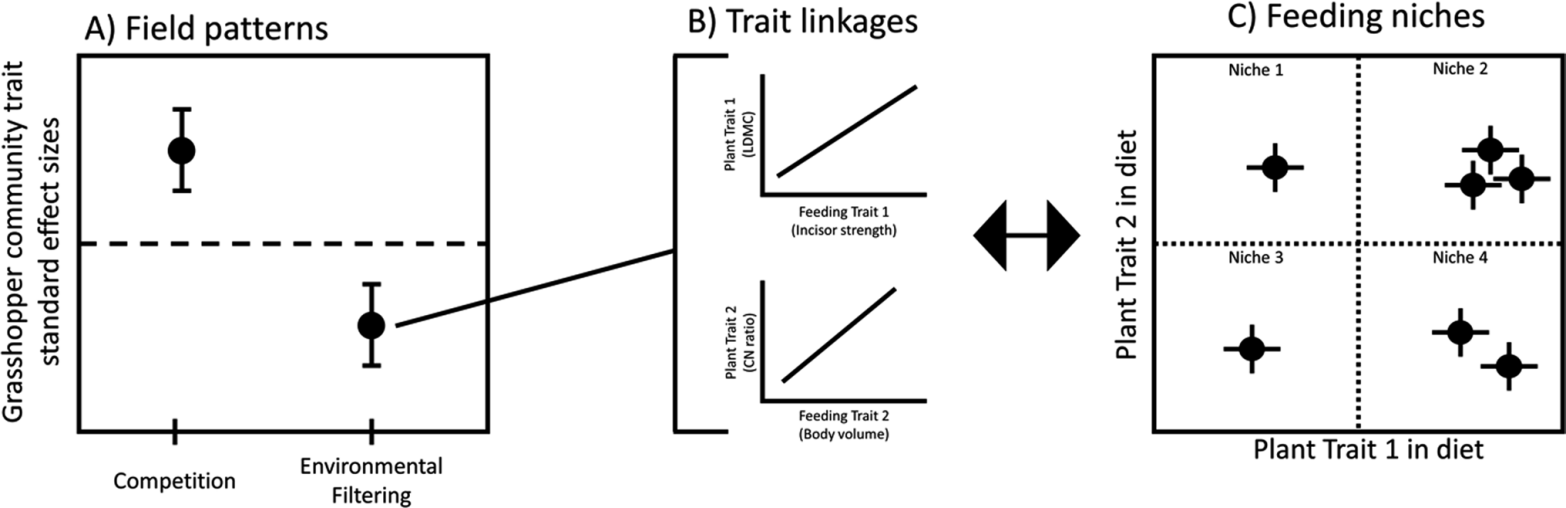
Predicted outcomes of community sampling and feeding assays in our study. Panel A represents the expected trends of the herbivore community trait standard effect sizes for niche differentiation driven by interspecific competition (represented by an over-dispersion pattern) or environmental filtering by the plant community (represented by an under-dispersion pattern). Panel B represents the expected correlations between herbivore feeding traits and plant traits incorporated into their diet. Strong correlations indicate ‘trait matching’ between the plants and herbivores. Panel C represents the possible results from our analysis of herbivore feeding niches (i.e., diet breadth) defined by the plant traits incorporated into their diet. Points represent individual grasshopper species

In this study, we combined a variety of methods using functional traits to examine the relationships between: (1) the traits and co-occurrence patterns of different insect herbivore species within communities in the field and (2) insect herbivore feeding preferences using choice experiments. First, we identified correlations between insect herbivore functional traits to establish which traits contribute to life history trade-offs and possibly play a role in herbivore niche partitioning. Second, we used insect functional traits to explain the community assembly patterns of insect herbivores in the field. In our system, if competition for food resources is strong, we expect grasshopper feeding traits to be over-dispersed in the field. If site-level characteristics, such as the trait composition of the plant community, filter grasshopper communities, we expect grasshopper feeding traits to be under-dispersed. Finally, we quantified herbivore feeding niches based on the plant functional traits incorporated into their diet and then established trophic linkages between herbivores and plants by identifying correlations between functional traits of the respective taxa. We did this by evaluating the feeding preferences of insect herbivores on a variety of plant species in cafeteria-style mesocosm experiments. We predicted that incisor strength would be positively correlated with a plant toughness metric like leaf dry matter content (LDMC) (Perez-Harguindeguy et al. 2016). Additionally, we hypothesized that herbivore nutritional requirements may be based on body size or nutritional status, so we predicted that herbivore body volume or C:N ratio would be correlated with plant C:N ratio (Fig. 1B). We also evaluated the feeding niche of grasshoppers based on the range of plant traits they fed upon. Grasshopper species that preferentially incorporate significantly different plant trait values into their diet will fill separate dietary niches. Grasshopper species that preferentially feed on plants with similar traits would fill a similar dietary niche and likely experience competition or coexist through neutral processes (Fig. 1C). Understanding functional trait relationships between trophic levels will provide a better understanding of the specific mechanisms governing insect herbivore community assembly and niche differentiation.

## Methods

### Study site

Community sampling was conducted at Ordway-Swisher Biological Station in Melrose, FL (Putnam Co., N29°41’, W82°). Nearly all plant and insect species used in this study were collected at this station, with some supplemental collection in a similar pine savanna site at the Natural Area Teaching Laboratory (NATL) on the University of Florida campus in Gainesville, FL (Alachua Co.). Sampling occurred in upland pine savanna in North Central Florida. Our sites were primarily xeric with a canopy of widespread longleaf pine (*Pinus palustris*) with occasional clusters of turkey oak (*Quercus laevis*). The understory was host to a highly diverse array of plant species (Jose et al. 2006) but is dominated by bunchgrasses (*Aristida beyrichiana* and *Sporobolus junceus*) (Appendix 1, Fig. S1).

### Herbivore study species

We used fourteen grasshopper species, spanning four subfamilies and three functional feeding categories (Appendix 1, Table S1). These categories included (1) grass-feeders (subfamily Gomphocerinae), (2) forb-feeders (subfamily Melanoplinae), and (3) mixed-feeders (subfamilies Cyrtacanthacrididae and Oedipodinae) (Joern 1979; Masloski et al. 2014).

### Herbivore trait measurements

Three functional traits were measured on adult individuals of 14 grasshopper species: body size, incisor strength, and C:N ratio. For each grasshopper species, we aimed to measure 10 males and 10 females (*n* = 20 individuals measured per species). All measurements were taken in millimeters. Body size and incisor strength were measured based on the existing protocols described in Moretti et al. (2017). C:N ratio was measured as detailed in Deraison et al. (2015a). Traits were categorized based on their relation to specific physiological functions (e.g., incisor strength = feeding trait).

Incisor strength is a measure of bite force and was calculated using a lever equation and measurements based on Ibanez et al. (2013):

$$Incisor \, strength \, = \, F_{in} L_{in} / \, L_{out} \times \, 1/R_{i}$$

where *F*_*in*_ is the strength of the grasshopper mandibular adductor muscle (head volume was measured as a proxy), *L*_*in*_ is the length (mm) between the adductor muscle point of attachment and the mandible axis of rotation, and *L*_*out*_ is the length (mm) between the incisive region and the mandible axis of rotation (Appendix 1, Fig. S2). Previous research has shown that incisor strength can be used to predict the toughness of plant material incorporated into the diet (Ibanez et al. 2013), and mean incisor strength of grasshopper communities can influence primary productivity (Deraison et al. 2015a). Mandibles of the grasshoppers were removed using precision tipped forceps and photographed using an Aven Mighty Scope 5 M Digital Microscope and EZ Image X2 software (Appendix 1, Fig. S2). Measurements were taken using ImageJ software.

Body size of grasshoppers is related to the nutritional quality of food and the amount of food incorporated into the diet (Yang and Joern 1994) and is calculated as body volume (Moretti et al. 2017) using the cylinder equation:

$$Volume \, = \, \left( {\pi /4} \right) \, LD^{2}$$

where *L* is the length of the body (head to tip of the abdomen), and *D* is the mean diameter of the body taken at four points on the grasshopper (head, thorax, base of the abdomen, and tip of the abdomen).

C:N ratio was considered a nutritional trait. Grasshoppers were dried at 50 °C for at least 24 h in preparation for nutritional analysis. Grasshopper heads were excised and placed in 2 mL screw-top centrifuge tubes with three zirconium beads. Tubes were then placed in a bead homogenizer until the tissue was a fine powder. Samples were sent to the University of Florida Stable Isotope Laboratory for analysis using flash combustion on a Costech 4010 Elemental Combustion System.

### Herbivore community sampling

Insect communities were sampled at 30 sites within Ordway-Swisher Biological Station in North Central Florida, USA (29.72, − 82.00) during 2022. Sites were selected across a gradient of prescribed fire frequency ranging from two to sixteen burns since 1983. Time since the last fire at sites ranged from 28 to 3505 days. Each site was sampled at two time points, July and September. Sampling occurred over the course of two weeks for each time point. At each site, insects were collected by sweep-netting the understory vegetation in four 5 × 5-m plots for two minutes each. Samples were temporarily stored on ice until returned to the laboratory where they were stored in − 20 °C freezers. All sweeping was conducted between 09:00 and 15:00 on sunny days. Adult Orthopterans in the family Acrididae were identified to species using available dichotomous keys and field guides (e.g.,Capinera et al. 2001, 2004; Smith et al. 2004).

Orthopteran nymphs cannot easily be identified to the species level using dichotomous keys or field guides and are difficult to distinguish visually. Similar nymphs were grouped based on shared morphological characteristics and assigned a morphospecies code to denote nymphs of that species. A subsample of nymphs (3 or more) assigned to a morphospecies was subjected to DNA sequencing for final identification. A detailed description of our DNA sequencing methodology can be found in Appendix 2.

A total of 2679 individuals were collected; 606 adults, 1911 nymphs, and 162 unidentifiable specimens; the last were not included in the analysis.

### Mesocosm experiment

#### Plant species and trait measurements

Sixteen plant species were used in this study, representing eight families and three functional categories (Appendix 1, Table S1), including 3 grasses, 11 herbaceous forbs and legumes, and 2 woody plants (i.e., trees and shrubs). The plants used were chosen based on a combination of their high abundances at our field sites and unique array of trait values. Therefore, these plants represent the spectrum of food types known to be eaten by grasshoppers (Joern 1979). All plant leaves were collected from wild populations (Ordway-Swisher or, in some cases, NATL) from July through September of 2022. All traits were measured on ten individual plants per species and then averaged across that species. The traits selected for this study included leaf dry matter content (LDMC), specific leaf area (SLA), and C:N ratio. LDMC, or the inverse of leaf water content, is a metric that relates to the toughness and lifespan of a leaf (Weiher et al. 1999; Perez-Harguindeguy et al. 2016). SLA is a metric that relates to a leaf’s growth rate and photosynthetic capacity and is negatively correlated with leaf toughness (Weiher et al. 1999; Perez-Harguindeguy et al. 2016). C:N ratio represents a measure of the nutritional quality of leaf tissue (Perez-Harguindeguy et al. 2016). LDMC and SLA were measured according to the protocol in Perez-Harguindeguy et al. (2016). For C:N ratio, homogenized samples of plant tissue were sent to the University of Florida Stable Isotope Laboratory for analysis using flash combustion on a Costech 4010 Elemental Combustion System.

#### Mesocosm design

Mesocosms were staged in 30 × 30 × 30 cm insect rearing cages (Appendix 1, Fig. S3). For the duration of each mesocosm trial, ambient conditions were maintained at 25 °C and 60% RH in a climate-controlled rearing room. A 14:10 h day/night cycle was maintained using fluorescent lights (6000 K). Cages were stocked with a 20 × 20 × 5 cm plastic tray filled with sand as a medium to support the plant cuttings used in the trial. We collected plant cuttings of the 16 species from populations growing naturally in the field two days before the start of each trial. Cuttings were kept fresh in floral water tubes. Any pre-existing damaged plant material was removed before the start of the experiment, so only undamaged leaves were available. Plant cuttings were arranged in the sand medium in a 4 × 4 grid in randomized positions (Appendix 1, Fig. S3). Grasshoppers were collected a day prior and starved overnight before placement in the mesocosm trials.

#### Mesocosm assay

We implemented a cafeteria-style assay to assess the feeding preferences of 11 species of grasshoppers on the 16 plant species (Appendix 1, Table S1). Prior to experimentation, pre-feeding plant assessments were conducted for comparison to post-feeding damage assessments, which involved counting the number of leaves and measuring stalk length of the plant cuttings. A single adult grasshopper was then placed in mesocosms and allowed to feed freely for 48 h before removal. Floral tubes were refilled with tap water as needed throughout the trial to prevent the plant cuttings from drying out. Post-feeding damage assessments were conducted using a centimeter squared grid to approximate the amount of plant tissue removed during the trial. The amount of leaf area consumed during the trial was recorded to the nearest 0.05cm^2^. If a grasshopper died during the trial, that mesocosm was removed from the study. A total of 123 mesocosms were tested in this experiment, with 11–12 replicates per species (except one species with only 7 replicates due to unusually high mortality of males).

### Statistical analysis

All statistical analyses in this study were conducted using programming in R Version 4.3.0 (R Core Team 2023).

### Herbivore trait correlations

To understand the relationships of various herbivore functional traits, we conducted pairwise correlations among our three focal traits and, to aid in visualization, a principal component analysis (PCA). Trait values were averaged across individuals of each of the 14 grasshopper species. Incisor strength and body volume were log-transformed to fit a Gaussian distribution. Pairwise correlations were conducted using Pearson correlations. A PCA of all three grasshopper traits was then calculated using the prcomp() function in base R (R Core Team 2023) and visualized using the fviz_pca_biplot() function in the *factoextra* R package (Kassambara and Mundt 2020).

### Community trait dispersion patterns

To test for trait over-/under-dispersion based on co-occurrence patterns in the field, we calculated standardized effect sizes (SES) (Cadotte and Tucker 2017) using a null modeling approach. We first used a site-by-species matrix (*n* = 30 sites × 2 sampling points = 60 rows with 14 grasshopper species) to calculate Rao’s Functional Dispersion, which is a metric to estimate functional dispersion of traits within a community (Botta‐Dukát and Czúcz 2016). To generate null communities for comparison, we shuffled the row headers (i.e., grasshopper species) of the species x trait matrix. This approach randomizes species’ traits while keeping abundances and species richness constant within each site (Ricotta and Moretti 2011; Botta‐Dukát and Czúcz 2016). We created 999 simulated communities and calculated the mean and standard deviation of Rao’s functional dispersion for each row. Then we calculated SES for each row as follows: SES = (μ_OBS_ – μ_NULL_)/σ_NULL_. We calculated SES values for all traits combined, and separately for each trait: body size, incisory strength, C:N ratio, and subfamily. Subfamily was included to determine if there is a phylogenetic signal, at the level of subfamilies, in the community assembly patterns. While this approach does not quantitatively account for phylogenetic distances, it does account for qualitative phylogenetic differences, which can be used as a proxy when a phylogeny is not available (Tiede et al. 2018) as is the case for our species. We then extracted the SES values and compared them among the two sampling periods (July and October) and between the traits using a linear mixed model using the glmmTMB package (Brooks et al. 2017). The SES values were the response variable. Fixed effects were sampling period, trait type (all combined, body size, incisor strength, C:N, and subfamily), and their interaction. We included site and trait type nested within site as random effects to account for the multiple subsamples within site and the multiple trait types. We used the Anova() function from the car package to evaluate the significance of fixed effects (Fox and Weisberg 2019). To compare tests for trait over-/under-dispersion, we compared the SES values for each trait type to zero using the emmeans() function in the emmeans package with ‘infer = TRUE’ (Lenth 2022).

### Feeding niche based on plant functional traits

To determine the grasshopper feeding niches based on the traits of the plants they consume, we calculated the weighted mean plant functional trait values consumed by grasshoppers in the mesocosms using the functcomp() function in the *FD* R package (Laliberté et al. 2014). The weighted mean functional trait in the diet was calculated as community weighted means (CWM), which is commonly used in community ecology studies when sampling species at different sites (Ricotta and Moretti 2011):

$$CWM= {\sum }_{n=i}^{S} pij\times (trait)i$$

In our case, the amount of plant tissue consumed of each plant species (*S*) was considered the abundance (*pi*) for each individual grasshopper (i.e., “site”; *j*). Using the formula above then yields the weighted mean plant functional trait incorporated into the diet of each individual grasshopper. We did this for each of the three plant functional traits (LDMC, SLA, and C:N).

A MANOVA was run to test the significance of feeding differences of grasshopper subfamilies and individual species with regard to the weighted mean plant traits incorporated into the grasshopper’s diet. In this model, the response matrix was the weighted mean trait values incorporated into the diet (CWM LDMC, CWM SLA, and CWM C:N). The predictor variables were grasshopper species (*n* = 14 species) and grasshopper feeding guild (*n* = 4 subfamilies). Following the omnibus MANOVA, we conducted univariate ANOVAs for each plant trait. For visualization, the weighted mean values for each plant trait were averaged across each grasshopper species and then plotted in a two-dimensional plant trait space along with standard error. These plots of the range of plant trait values were used to understand how grasshoppers differentiated their niches based on different plant traits in their diet. Comparisons were made of each plant trait combination. To aid in interpretation of the feeding niche, we also plotted the ellipses around the plant trait values offered in each arena (*n* = 16 plant species).

Linear models were used to test for functional linkages of grasshopper and plant traits. These were constructed using the glmmTMB() function from the *glmmTMB* R package (Brooks et al. 2017). Mean grasshopper trait values were set as predictors of CWM plant trait values incorporated into the diet from the above calculations. We also included subfamily as a random effect to account for differences among subfamilies. Correlations were tested only for hypothesized trait linkages (see Introduction; Fig. 1B).

## Results

### Herbivore trait correlations

**Fig. 2:**
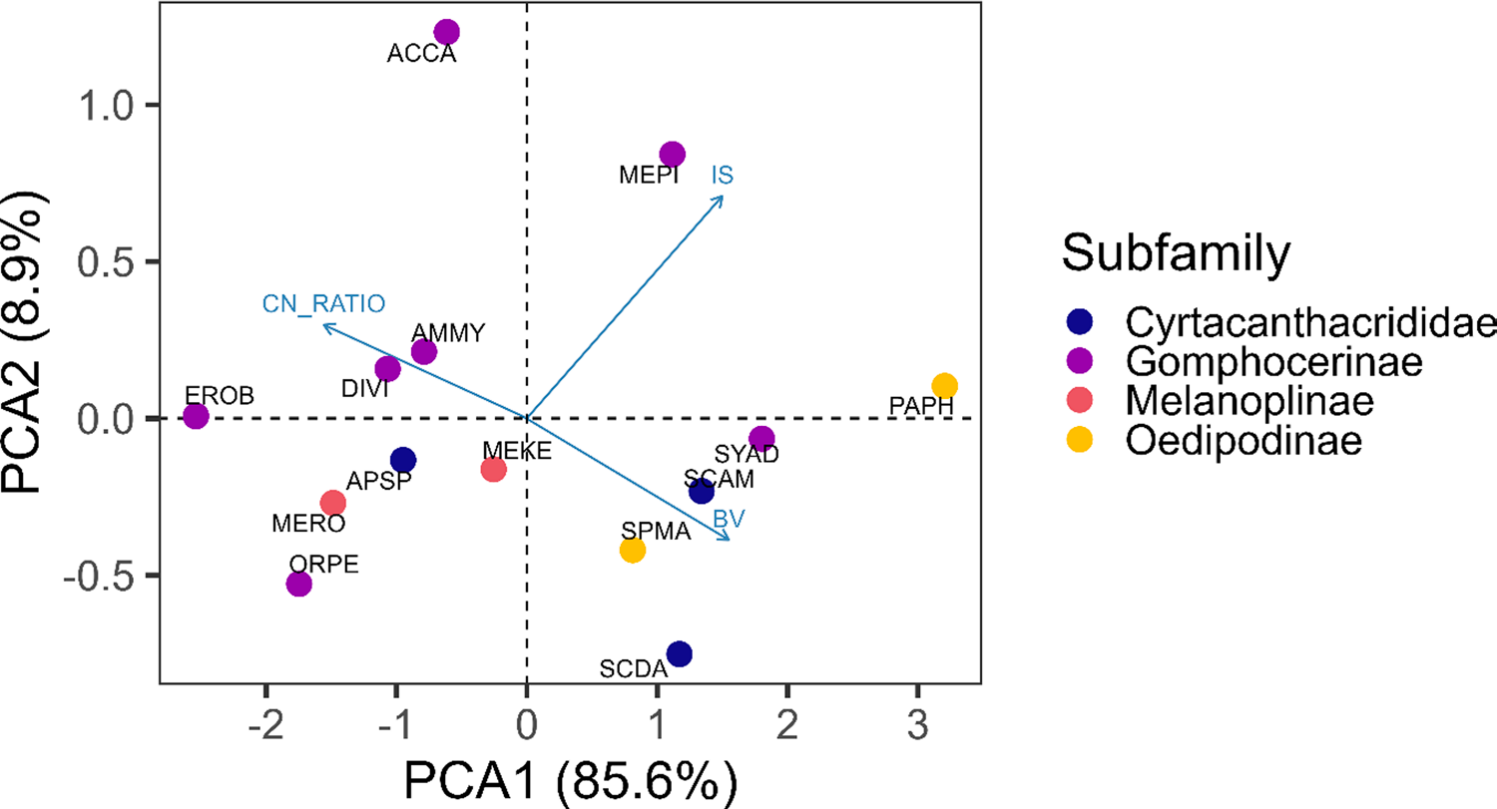
Principal component analysis (PCA) representing the relationship between grasshopper body volume (BV), incisor strength (IS; feeding trait), and C:N ratio (CN_RATIO; nutritional trait) measured on 14 grasshopper species. Points represent grasshopper species and are colored based on subfamily; codes indicate the first two characters of the genus and specific epithet

All three of the traits we measured were highly correlated. Grasshopper incisor strength and C:N ratio were negatively correlated (*r* = − 0.69, *p* = 0.009), incisory strength and body size were positively correlated (*r* = 0.78, *p* = 0.001), and C:N and body size were negatively correlated (*r* = − 0.74, *p* = 0.009). The first two principal components in the PCA described 94.6% of all variations between grasshopper traits. The first PC axis explained 85.6% percent of the variation, suggesting strong correlations among these three traits. Body volume and incisor strength loaded positively on the first axis, while C:N ratio loaded negatively. The second axis explained 8.9% of the variation where body volume loaded negatively while incisor strength and C:N ratio loaded positively (Fig. 2).

### Community trait dispersion patterns

**Fig. 3:**
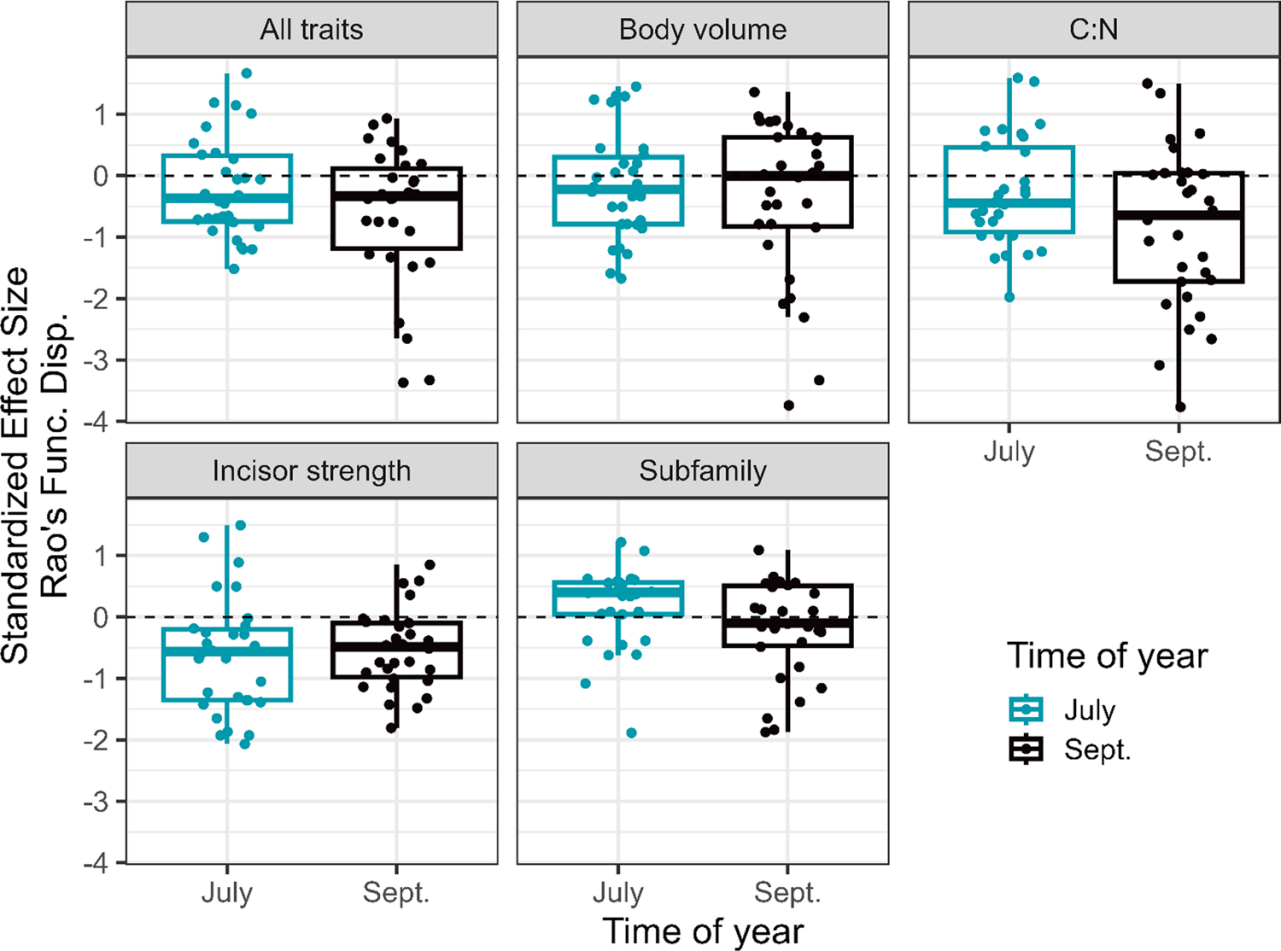
Graphs show the standardized effect size (SES) calculated using Rao’s Quadratic Entropy as a measure of functional dispersion. SES were calculated as Rao’s observed value minus the mean of Rao’s Q from a null model that randomized columns divided by the standard deviation of Rao’s Q from the null model simulations. Negative values indicate “under-dispersion” or trait-clustering, whereas positive values indicate trait over-dispersion

In our linear mixed model, SES values were significantly different between sampling season (χ^2^ = 8.9, df = 1, *p* = 0.003) and trait type (χ^2^ = 35.6, df = 4, *p* < 0.001), but the interaction was marginally significant (χ^2^ = 8.0, df = 4, *p* = 0.095). Pooled across the sampling periods, all traits combined showed under-dispersion (i.e., clustering) in Rao’s functional dispersion relative to null communities (mean SES = − 0.46 ± 0.13 SE; test ≠ 0: *t* = − 3.5, *p* = 0.0005). For each individual trait (Fig. 3), incisory strength showed the strongest degree of under-dispersion (mean SES = − 0.60 ± 0.12 SE; test ≠ 0: *t* = − 4.9, *p* < 0.001), followed by C:N ratio (mean SES = − 0.59 ± 0.15 SE; test ≠ 0: *t* = − 4.1, *p* = 0.001), and then body volume (mean SES = − 0.28 ± 0.15 SE; test ≠ 0: *t* = − 1.9, p = 0.061). The subfamily effect did not differ from the null communities (mean SES = 0.06 ± 0.12 SE; test ≠ 0: *t* = 0.5, *p* = 0.61), suggesting limited phylogenetic signal in assembly patterns. Patterns were also generally stronger in the early season (mean SES = − 0.22 ± 0.11 SE; test ≠ 0: *t* = − 2.1, *p* = 0.04) compared to late season (mean SES = − 0.53 ± 0.11 SE; test ≠ 0: *t* = − 5.0, *p* < 0.001), pooled across all traits.

### Herbivore feeding niches

**Fig. 4:**
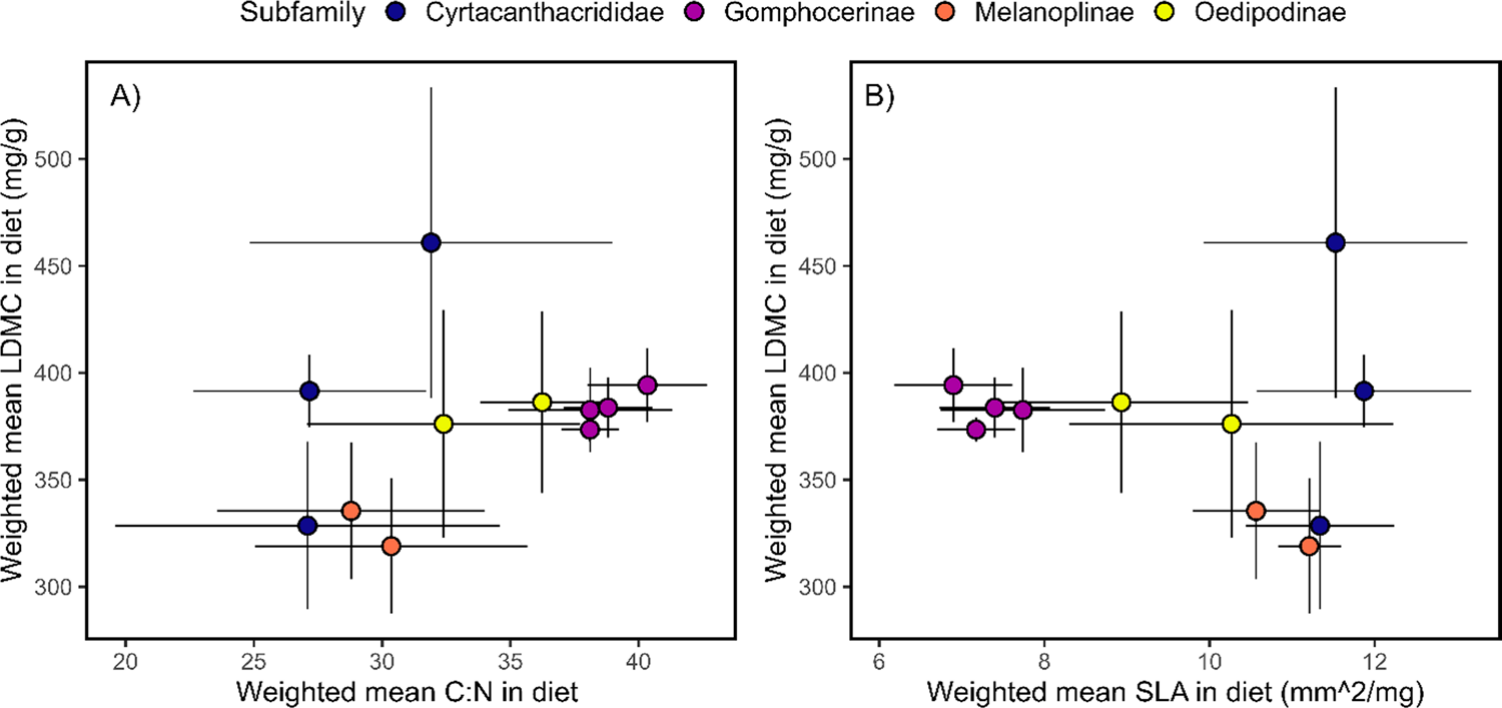
Feeding niches of grasshoppers plotted in trait space based on the weighted mean of A) C:N ration and LDMC and B) SLA and LDMC incorporated into the grasshopper’s diet. Points represent individual grasshopper species and are colored based on grasshopper subfamily. Bars represent the range of plant trait values incorporated into the diet (1 standard deviation; ~ 12 individual grasshoppers tested per species)

MANOVA revealed that grasshopper diets differed between subfamilies (approx. *F*_9,336_ = 19.8, *p* < 0.001) and between individual grasshopper species (approx. *F*_21,336_ = 3.7, *p* < 0.001) based on all plant traits incorporated into their diets. The same trend was observed when evaluating individual plant traits incorporated into the diets, using ANOVA, between subfamilies and grasshopper species (Appendix 1, Table S2). The effect between species for SLA and plant C:N ratio incorporated into the diet only differed with marginal significance between species (Appendix 1, Table S2). For all plant trait spaces, grasshopper diets were clustered based on subfamily, with Cyrtacanthacrididae having the most variable diet within a subfamily (Fig. 4).

### Plant–Herbivore trait-matching

**Fig. 5:**
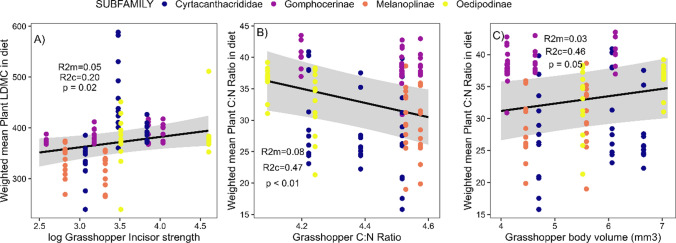
Trait linkages between grasshopper functional traits and the weighted mean of plant functional traits incorporated into the diet. **A** relationship between grasshopper incisor strength and plant LDMC, **B** relationship between grasshopper C:N and plant C:N ratio, and **C** relationship between grasshopper body volume and plant C:N ratio

Grasshopper incisor strength and LDMC were positively correlated (χ2 = 5.0, df = 1, p = 0.025, R^2^m = 0.05, R^2^c = 0.20; Fig. 5A). Grasshopper C:N ratio and plant C:N ratio were negatively correlated (χ2 = 8.3, df = 1, *p* = 0.004, R^2^m = 0.08, R^2^c = 0.47; Fig. 5B). Similarly, grasshopper body volume and plant C:N ratio were positively correlated (χ2 = 3.8, df = 1, *p* = 0.049, R^2^m = 0.03, R^2^c = 0.46; Fig. 5C).

## Discussion

A longstanding question in ecology asks how species in diverse communities partition resources to coexist. Reimagining the niche concept in functional trait space has been particularly effective for explaining patterns of plant community assembly across environmental gradients (McGill et al. 2006; Violle and Jiang 2009). Our research significantly contributes to this idea by providing evidence that insect functional traits may elucidate the underlying mechanisms that govern community assembly processes across environmental gradients. In our case, we found that grasshopper communities tended to exhibit under-dispersion (i.e., clustering) for traits related to feeding (incisor strength). Further, our lab experiments advance our understanding of the ecological importance of functional traits by showing that insect functional traits clearly influence insect herbivore diet niche differentiation and that insect feeding traits are functionally linked to plant traits related to toughness. This research presents a strong case for a broader implementation of insect functional traits to address fundamental and applied questions in community ecology research.

Functional traits are often highly correlated, either positively due to similar functionality or negatively due to physiological constraints or life history trade-offs (Flatt and Heyland 2011). We examined the correlations among numerous functional traits in grasshoppers, an ecologically important taxon (Rodell 1977; Belovsky and Slade 2018), but one that has been understudied in terms of functional traits (but see Ibanez et al. 2013; Deraison et al. 2015a, b; Neff et al. 2019). The principal component analysis of the three grasshopper feeding/nutritional traits showed strong correlations. Lower grasshopper C:N ratio represents a high nutritious quality, which was correlated with stronger incisors and larger body volume (Fig. 2). This could suggest a trade-off where larger and stronger feeding grasshoppers require higher nitrogen inputs than weaker feeding species to maintain a more costly feeding strategy. Alternatively, this could suggest that stronger feeding species can access more nitrogen-rich resources due to an increased pool of dietary options. Regardless, our research documents the potential for feeding strategies (or syndromes) and provides an avenue for future investigation of the ecological and evolutionary consequences of these feeding strategies (Bakewell et al. 2020).

### Community trait dispersion patterns

Trait dispersion in the field exhibited weak but consistent under-dispersion for most traits, suggesting that these communities are structured by environmental filtering. Although experiments have revealed that grasshoppers are often food-limited and therefore compete for food between species (Ritchie and Tilman 1992; Belovsky and Slade 1993), they can shift their feeding behavior when co-occurring with other species to limit direct competition for food (Ritchie and Tilman 1992; Chase 1996; Beckerman 2000). The trait with the strongest under-dispersion was incisor strength, followed by grasshopper C:N ratio, and body volume (Fig. 3). This result suggests that site characteristics, such as time since fire or plant community composition, are filtering grasshoppers based on their functional traits. Subfamilies did not show any dispersion pattern (mean SES = 0.02 ± 0.14SE), suggesting that the trait dispersion patterns were not driven by a phylogenetic pattern of closely related species being more likely to co-occur than unrelated species, at least at the subfamily level. Therefore, the most plausible explanation is that environmental filtering occurs because grasshopper species map onto plant communities based on the grasshopper’s functional traits rather than communities being structured by competition. Although these results are consistent with a previous study that combined field surveys with competition experiments (Beckerman 2000), interpreting observation community patterns by themselves is notoriously difficult (Cadotte and Tucker 2017; Lepš and De Bello 2023). For example, an alternative explanation is perhaps equally as likely. Plant communities recovering from fire tend to have more acquisitive traits, such as higher SLA, lower LDMC, and lower C:N ratio (Archibald et al. 2019; Mitchell et al. 2021), all of which tend to be more palatable to grasshoppers. Since most grasshopper species respond by increasing in abundance shortly after a fire (Joern 2005; Hahn and Orrock 2015b), it is likely that niche partitioning is still important for maintaining diverse communities, even given this filtering effect. In other words, grasshopper species may all respond to increased resource quality, while still competing to some degree for shared resources. An important limitation of this study is that plant community data were not collected, and therefore could not explicitly test the hypothesized direct relationship between plant and grasshopper communities. Nevertheless, because previous studies have documented indirect effects of fire on grasshopper abundance and taxonomic diversity, mediated through changes in the plant communities (Hahn and Orrock 2015a, b; Miller et al. 2017), these effects likely influence grasshopper community traits.

### Feeding niches and trait-matching

Our mesocosm experiments further support the notion that grasshoppers limit direct competition for food resources, specifically through differentiation of their dietary niches. We found strong evidence that insect herbivores differentiate their feeding niches based on which plant traits they incorporate into their diets. Grasshopper feeding preferences primarily differentiate based on their subfamily, which correlates strongly with their feeding guild (Joern 1979; Masloski et al. 2014). Subfamilies differed highly by the LDMC, SLA, and C:N ratio of the plants they preferentially fed upon. To a lesser degree, grasshoppers also differentiated based on species, with the LDMC incorporated in the diet being highly differentiated most strongly between species and, to a lesser degree, SLA and C:N ratio. This suggests that within subfamilies, there may be a higher occurrence of competition due to increased niche overlap (Fig. 4A, B). Alternatively, species within subfamilies may coexist through neutral processes (i.e., being sufficiently similar; Scheffer et al. 2018), or may partition their niches more subtly based on processes related to morphology or unmeasured characteristics such as protein or carbohydrates (Behmer and Joern 2008). One caveat of our experiment is that mesocosms were choice tests containing only one grasshopper at a time. Thus, dietary preferences could be considered the “fundamental” feeding niche, at least given the plants that were offered in the experiment. Dietary preferences likely change when competing with other species (Ritchie and Tilman 1992; Chase 1996), which deserves further testing. Nevertheless, even when alone, we did see feeding differences both among subfamilies and to a lesser degree among species within subfamilies (Fig. 4). This finding contributes to understanding how different factors may operate at different scales to structure grasshopper communities. Results from our field surveys showed that incisor strength tended to be under-dispersed, suggesting that there is environmental filtering occurring at the site level. However, within sites, grasshopper species with similar traits can avoid competition by preferentially feeding on different plants, which would be a mechanism that would maintain coexistence.

Trait-matching between the two trophic levels was observed for herbivore feeding and plant toughness traits. The strongest trait-matching we observed was that grasshoppers with stronger incisors preferentially fed on plants with higher LDMC (Fig. 5A). It is likely that weaker feeding grasshoppers capitalize on faster-growing plant species with lower defenses (Coley et al. 1985; Price 1991; Čížek 2005), relegating stronger mandible species to selectively feed on tougher plant material due to the inaccessibility of tougher plants to weaker feeding grasshoppers. This would result in strong niche differentiation and enable coexistence, which is also consistent with the feeding niche results, showing that grasshopper species differentiate their feeding niches based on LDMC (Fig. 4). Changes in grasshopper incisor strength likely mediate this differentiation; however, due to the limitations of our study design, it is difficult to determine if plant trait values govern grasshopper dietary selection or if an unknown plant factor correlating with LDMC is driving grasshopper feeding preferences. A biomechanical study testing different grasshopper feeding preferences on artificial diets with differing toughness could provide evidence to make a concrete conclusion matching grasshopper incisor strength to plant LDMC. Similarly, trait-matching was also observed between grasshopper C:N ratio and the C:N ratio of plants consumed. However, this correlation was negative and relatively weak, where grasshoppers with lower C:N ratio tended to consume plant species with higher C:N ratio (and vice versa; Fig. 5B). Similarly, smaller-bodied species tended to consume plants with higher C:N ratios (Fig. 5C). Because lower C:N grasshopper species tended to be larger bodied (Fig. 2), it is likely they were meeting their nutritional requirements by consuming larger amounts of food and decreased metabolic rate of increased body size (Yang and Joern 1994).

Plant–herbivore interactions are complex processes that are likely governed by a complex of interacting traits between either trophic level. It is important to remember that our analysis tested for strong correlations between individual grasshopper and plant traits. As was mentioned previously, functional traits are often correlated (Flatt and Heyland 2011). Strong correlations between the traits tested here, such as that between incisor strength and LDMC, represent traits that most strongly govern the interactions between the plants and herbivores. Still, it is highly plausible that other traits contribute to the likelihood of these interactions. Additionally, trait values for each grasshopper species were averaged across all tested individuals of a given species. This prevents us from examining intraspecific trait variation and how this impacts plant–herbivore interactions. Research examining the variability of trait values within a species of herbivore, whether traits are over- or under-dispersed within a species, and how individuals within a species differ in their dietary preferences regarding plant traits would provide a clearer understanding of the role traits play in determining plant–herbivore interactions, compared to other factors such as phylogeny.

## Conclusion

Our investigation of the relationships between insect herbivore functional traits uncovered links between herbivore feeding and nutrition that help describe the trait-clustering patterns for incisor strength that we documented in the field. Our research showcases the utility of insect functional traits as a tool for studying insect community dynamics and provide evidence that insect functional traits potentially govern community assembly patterns. Further, insect functional traits provide a clearer explanation of how insects interact with plants within communities. The trait-matching patterns, particularly for incisor strength and plant LDMC, and dietary niche differentiation help explain the trait-clustering patterns we saw in the field, thereby expanding our understanding of how closely related grasshopper species partition feeding niches to coexist (Behmer and Joern 2008). Further exploration into other insect functional traits, such as metabolic and consumption rates, and their relationship with leaf economic traits could provide additional ecologically meaningful insights into the niche differentiation patterns of insects that were not explored in this study. Additionally, considering intraspecific trait variation will elucidate the relative importance of traits compared to phylogeny in governing trophic interactions. Studying the traits of insects at different trophic levels and of different taxa will lead to a better understanding of insect community dynamics. This will potentially lead to the development of general rules in this area of research and subsequently make insect community composition and their impacts on ecosystem functioning more predictable. For example, supporting communities of insects with diverse trait values on agricultural land could potentially improve the functioning and productivity of farms through various mechanisms (Tamburini et al. 2020; Hahn and Cammarano 2023). Understanding how insect functional traits impact ecosystem functioning will ultimately inform management and conservation of natural and managed ecosystems.

## Supplementary Information

Below is the link to the electronic supplementary material.

Supplementary file1 (PDF 266 KB)

## Data Availability

We will deposit all data and code associated with the manuscript in Dryad upon acceptance.
