# Supplementary material for: Insect functional traits reveal processes that shape niche differentiation patterns
Source: Oecologia. 2025 Aug 21;207(9):147. doi: 10.1007/s00442-025-05783-4 (PMC12370786; doi:10.1007/s00442-025-05783-4)
Supplement: Supplementary file 1 — Supplementary file1 (PDF 266 KB) [file 442_2025_5783_MOESM1_ESM.pdf]

Supplementary Material

Appendix 1: Tables and Figures

Table S1. List of insect herbivore and plant species used in this study. *Indicates grasshopper species that were used in the mesocosm experiments.

Trophic Level	Functional Group	Family/Subfamily	Species
Herbivore	Grass-Feeders	Gomphocerinae	*Achurum carinatum**
			Amblytropidia mysteca
			*Dichromorpha viridis**
			Eritettix obscurus
			Mermiria picta
			*Orphulella pelidna**
			*Syrbula admirabilis**
	Mixed-Feeders	Oedipodinae	*Pardalophora phoenicoptera**
			*Spharagamon marmorata**
		Cyrtacanthacridinae	*Aptenopedes sphenarioides**
			*Schistocerca americana**
			*Schistocerca damnifica**
	Forb-Feeders	Melanoplinae	*Melanoplus keeleri**
			*Melanoplus rotundipennis**
Plant	Grass	Poaceae	*Aristida beyrichiana*
			Schizachyrium scoparium
			Sorghastrum secundum
	Forb	Euphorbiaceae	*Croton argyranthemus*
			Stillingia sylvatica
		Polygonaceae	*Eriogonum tomentosum*
		Apiaceae	*Eryngium aromaticum*
		Lamiaceae	*Monarda punctata*
		Asteraceae	*Ambrosia artemisiifolia*
			Pityopsis graminifolia
			Solidago odora
		Fabaceae	*Chamaecrista nictitans*
			Lespedeza hirta
			Tephrosia virginiana
	Woody	Fagaceae	*Quercus laevis*
		Ericaceae	*Vaccinium myrsinites*

Table S2. P-values from MANOVA used to evaluate herbivore feeding niche differentiation based on plant functional traits of plants consumed.

	Plant Functional Traits			
	LDMC	SLA	C:N Ratio	Full Model
Grasshopper Subfamily	<0.001	<0.001	<0.001	<0.001
Grasshopper Species	<0.001	0.073	0.07	<0.001

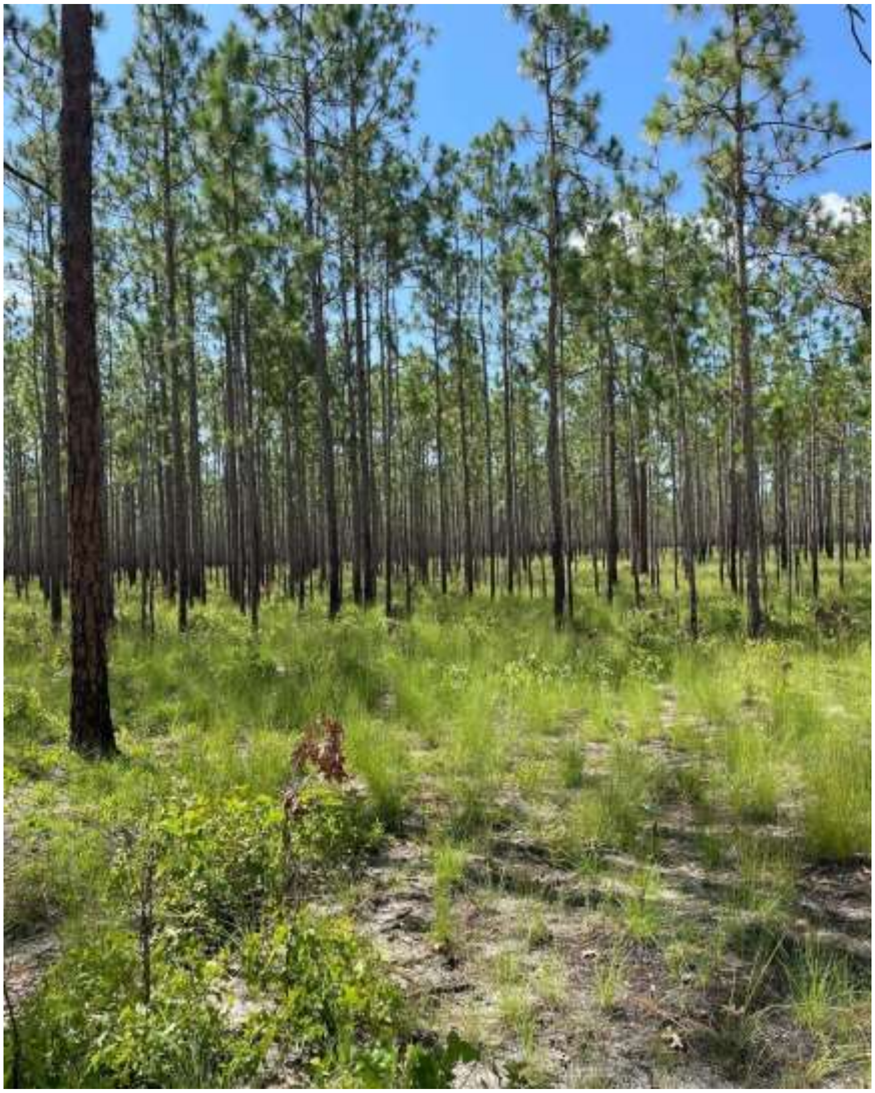

Figure S1. North Florida pine savanna habitat. Image was taken in July of 2022 at Ordway-Swisher Biological Station in Melrose, FL.

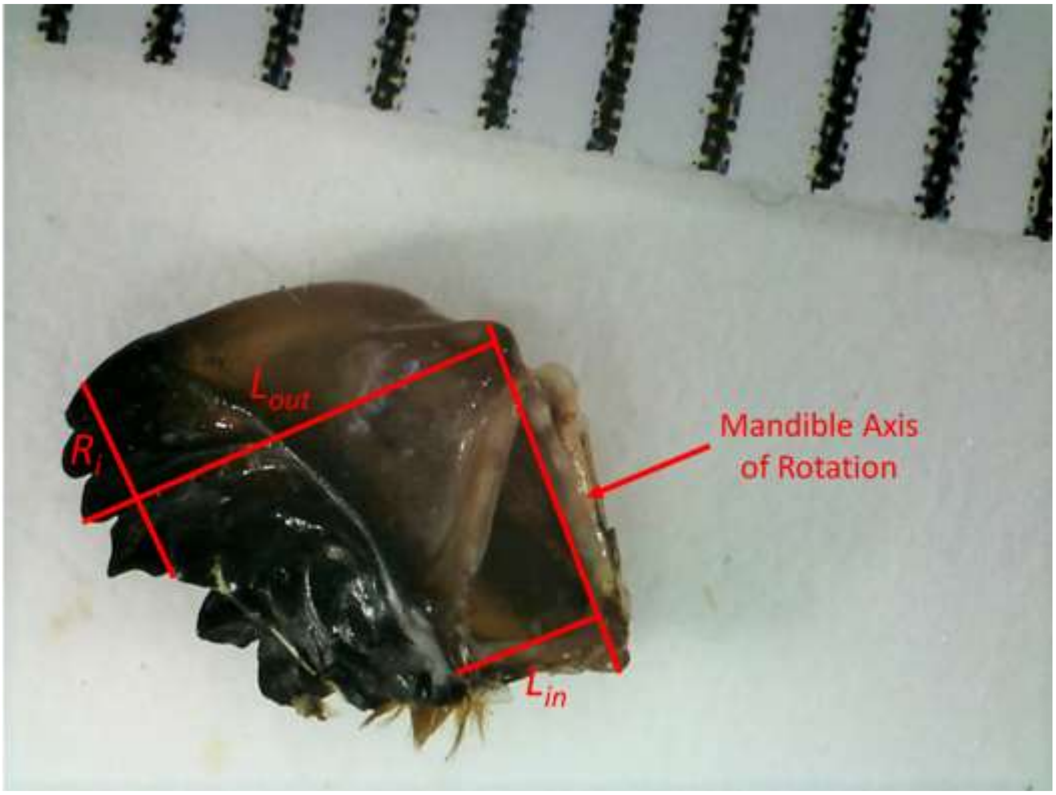

Figure S2. Grasshopper mandible with measurements for calculating incisor strength.

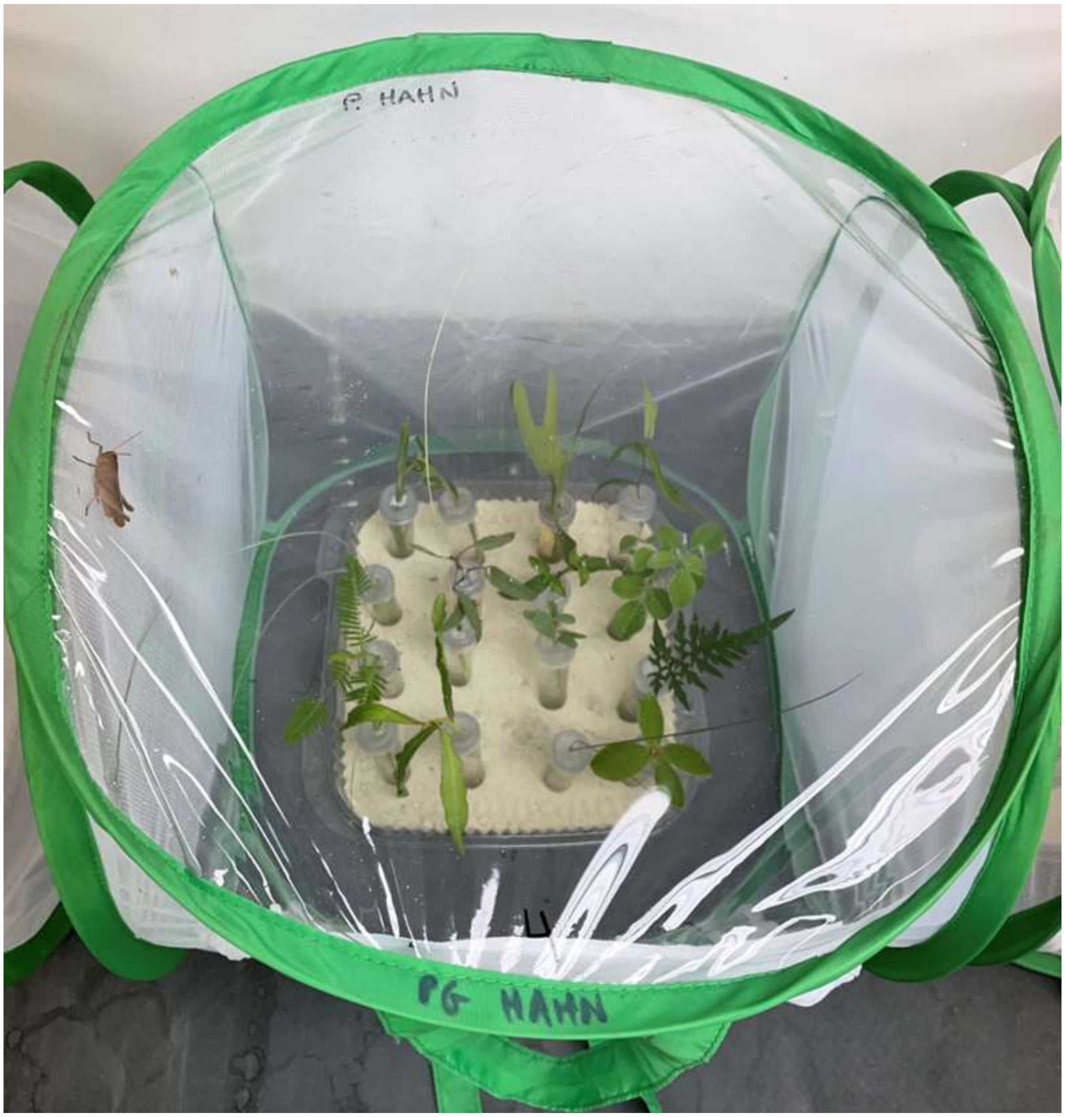

Figure S3. Mesocosm design for cafeteria style feeding assay.

Appendix 2: DNA Barcoding

Extraction. DNA extractions were carried out using materials from the Qiagen DNeasy® Blood and Tissue Kit. To start, 5-50 mg of insect tissue (1-2 forelegs depending on the size of the nymph) was placed in a 2 mL screw-cap microcentrifuge tube, along with 3 ceramic beads. To this, 100 µL of Hank's balanced salt solution (HBSS) was added. This mixture was homogenized using a bead beater for 1 minute, then an additional 80 µL of HBSS was added. To this, 20 µL of proteinase K was added. The mixture was then pulse-vortexed and left to incubate in a dry bath at 56°C for 10 minutes. Then, 200 µL of Buffer AL was added, vortexed, and incubated again at 56°C for 10 minutes. Next, 200 µL of 100% ethanol was added and the mixture was vortexed. The entire mixture was then transferred to a DNeasy spin column placed in a 2 mL collection tube. The column was then centrifuged at 8000 rpm for 1 min, and the flow-through and collection tube were discarded. The spin column was then placed in a new 2 mL collection and 500 µL of Buffer AW1 was added. The spin column was then centrifuged again at 8000 rpm for 1 minute and the flow-through and collection tube discarded. The spin column was then placed in a new 2 mL collection tube and 500 µL of Buffer AW2 was added. The spin column was then centrifuged at 14000 rpm for 3 min and the flow-through and collection tube was discarded. The spin column was then placed in a 1.5 mL microcentrifuge tube and 50 µL of Buffer AE was pipetted directly on to the spin column membrane. The membrane was left to incubate for 10 minutes at room temperature, then the spin column was centrifuged at 8000 rpm for 1 minute to elute the extraction. All extractions were stored at -80°C until ready for use.

Polymerase chain reaction. Polymerase chain reaction (PCR) was conducted to amplify our DNA extractions. The COBU/COBL primer pair described in Huang et al. 2013 was used for this reaction. These primers target the cytochrome oxidase 1 mitochondrial gene sequence and

are specifically optimized for Orthopteran DNA (Huang et al., 2013). Each reaction mixture included 12.5 μL of Thermo Scientific™ DreamTaq Green PCR Master Mix (2X), 0.5 μL COBU primer, 0.5 μL COBL primer, 10.5 μL of nuclease free water, and 1 μL of DNA extraction template. PCR mixtures were loaded into PCR strip tubes and centrifuged before thermocycling.

PCR tubes were loaded into a thermocycler with a lid temperature of 105°C. First, an initial denaturation was run at 95°C for 2 minutes. Then, denaturation occurred again at 95°C for 30 seconds. This was followed by annealing at 53°C for 1 min, then extension at 72°C for 1 minute. The 30 denaturation, annealing, and extension steps were repeated 35 times. The final extension step was run at 72°C for 10 minutes. The reactions were then left to idle at 4°C until collected.

Gel electrophoresis was run through a 1.5% agarose gel with added Thermo Fisher Scientific GreenGlo™ Safe DNA Dye. Gels were submerged in an electrophoresis buffer chamber filled with 1x TAE buffer. PCR reactions were loaded into gel wells and electrophoresis was run at approximately 100 volts for 30-60 minutes. Gels were placed on a UV transilluminator and base pair lengths of our PCR reactions were compared to a DNA ladder and a negative control. The reactions were stored at -20 to 4°C until sent for sanger sequencing.

Samples were sent to Genewiz from Aventa Life Science for sanger sequencing. DNA barcode sequences were then input into the NCBI BoLD and BLAST databases and compared to identified species. Percent identity results were cross-referenced between the two databases to determine a species match.

Huang, J., Zhang, A., Mao, S., & Huang, Y. (2013). DNA barcoding and species boundary delimitation of selected species of Chinese acridoidea (orthoptera: Caelifera). *PLoS ONE, 8*(12). https://doi.org/10.1371/journal.pone.0082400
